# Smartphone-Enabled, Telehealth-Based Family Conferences in Palliative Care During the COVID-19 Pandemic: Pilot Observational Study

**DOI:** 10.2196/22069

**Published:** 2020-10-28

**Authors:** Yu-Rui Wu, Tzu-Jung Chou, Yi-Jen Wang, Jaw-Shiun Tsai, Shao-Yi Cheng, Chien-An Yao, Jen-Kuei Peng, Wen-Yu Hu, Tai-Yuan Chiu, Hsien-Liang Huang

**Affiliations:** 1 Department of Family Medicine Taitung Christian Hospital Taitung Taiwan; 2 Department of Family Medicine National Taiwan University Hospital Taipei City Taiwan; 3 Department of Nursing National Taiwan University Hospital Taipei City Taiwan

**Keywords:** smartphone, mobile phone, telehealth, family conference, shared decision making, COVID-19, palliative care, end-of-life care

## Abstract

**Background:**

In the palliative care setting, infection control measures implemented due to COVID-19 have become barriers to end-of-life care discussions (eg, discharge planning and withdrawal of life-sustaining treatments) between patients, their families, and multidisciplinary medical teams. Strict restrictions in terms of visiting hours and the number of visitors have made it difficult to arrange in-person family conferences. Phone-based telehealth consultations may be a solution, but the lack of nonverbal cues may diminish the clinician-patient relationship. In this context, video-based, smartphone-enabled family conferences have become important.

**Objective:**

We aimed to establish a smartphone-enabled telehealth model for palliative care family conferences. Our model integrates principles from the concept of shared decision making (SDM) and the value, acknowledge, listen, understand, and elicit (VALUE) approach.

**Methods:**

Family conferences comprised three phases designed according to telehealth implementation guidelines—the previsit, during-visit, and postvisit phases. We incorporated the following SDM elements into the model: “team talk,” “option talk,” and “decision talk.” The model has been implemented at a national cancer treatment center in Taiwan since February 2020.

**Results:**

From February to April 2020, 14 telehealth family conferences in the palliative care unit were analyzed. The patients’ mean age was 73 (SD 10.1) years; 6 out of 14 patients (43%) were female and 12 (86%) were married. The primary caregiver joining the conference virtually comprised mostly of spouses and children (n=10, 71%). The majority of participants were terminally ill patients with cancer (n=13, 93%), with the exception of 1 patient with stroke. Consensus on care goals related to discharge planning and withdrawal of life-sustaining treatments was reached in 93% (n=13) of cases during the family conferences. In total, 5 families rated the family conferences as *good* or *very good* (36%), whereas 9 were *neutral* (64%).

**Conclusions:**

Smartphone-enabled telehealth for palliative care family conferences with SDM and VALUE integration demonstrated high satisfaction for families. In most cases, it was effective in reaching consensus on care decisions. The model may be applied to other countries to promote quality in end-of-life care in the midst of the COVID-19 pandemic.

## Introduction

Face-to-face communication is indispensable in palliative care settings, but the COVID-19 outbreak may strain this well-established way of delivering end-of-life family conferences. This emerging infectious disease has posed an unprecedented threat, devastating the economy and medical systems of countries worldwide [1]. Physicians and health care facilities are thus confronted with various challenges, including limited medical resources and capacities as well as corresponding restrictions. Restrictions in hospital visits and visiting hours, put in place to reduce the transmission of COVID-19, have become obstacles to arranging family conferences [2,3]. Fewer family members have access to hospitals for meetings with medical teams compared to prepandemic times. This has decreased chances for communication and interaction between clinicians and patients and their families.

Health care professionals face many challenges and ethical dilemmas when caring for terminally ill patients; finding the most appropriate management solution requires communication among patients, caregivers, and multidisciplinary medical teams [4,5]. Discharge planning and withdrawal of life-sustaining treatments are top-ranking ethical dilemmas in palliative care, especially among inpatients [6,7]. Decisions related to place of care at the end of life have become increasingly complicated during the COVID-19 pandemic due to worries about the disease’s high infection rate; however, families may encounter difficulties when caring for patients at home. In end-of-life care, it is our responsibility to realize the essence of shared decision making (SDM), which integrates the patient’s preferences with the best-known evidence [8,9]. Furthermore, exploring the family’s preferred role and desire is one of the key components of high-quality SDM, and thus incorporating the concept of SDM into family conferences is important [10]. Therefore, family conferences are an appropriate venue for engagement between all parties involved; here, patients and families can express their worries, receive integrated information from the medical team, and achieve concordance of caring goals.

The essence and main principle of palliative care is to relieve the physical and psycho-spiritual sufferings of the patient and their family, and to console their feelings. Previous studies have shown that not only terminally ill patients but their families as well suffer from physical and psychological distress, leading to subsequent comorbidity and increasing mortality [11,12]. On the other hand, the five principles of VALUE (value, acknowledge, listen, understand, and elicit) were proposed to improve physician-family communication, originally in intensive care units (ICUs), and has been shown to significantly decrease emotional disorders, such as posttraumatic stress disorder, in families even 3 months after a patient’s death [13,14]. However, in the clinician-patient relationship, nonverbal interactions and appearance cues play fundamental roles in successful communication [15,16]. The patient’s nonverbal reactions may be helpful to the physician for diagnosis and treatment decisions, and the clinician’s nonverbal behaviors are related to patient satisfaction [17]. The lack of nonverbal communication is one of the major drawbacks of telehealth and diminishes the clinician-patient relationship. Under the circumstances, the optimal way to adhere to infectious disease prevention measures and promote physician-patient relationship might be telehealth-based family conferences with the integration of appropriate communication strategies such as VALUE.

In theory, implementing the VALUE approach for family conferences on the basis of SDM should solidify communication between clinicians and families. In this study, we aimed to establish a model of smartphone-enabled telehealth for palliative care family conferences with SDM and VALUE integration in order to (1) increase access to communication with the clinician under the visitor restrictions imposed by the pandemic, (2) reach consensus on care goals, and (3) achieve patient and family satisfaction with telehealth-based conferences.

## Methods

### A Model for Telehealth-Based Family Conferences in Palliative Care

**Figure 1:**
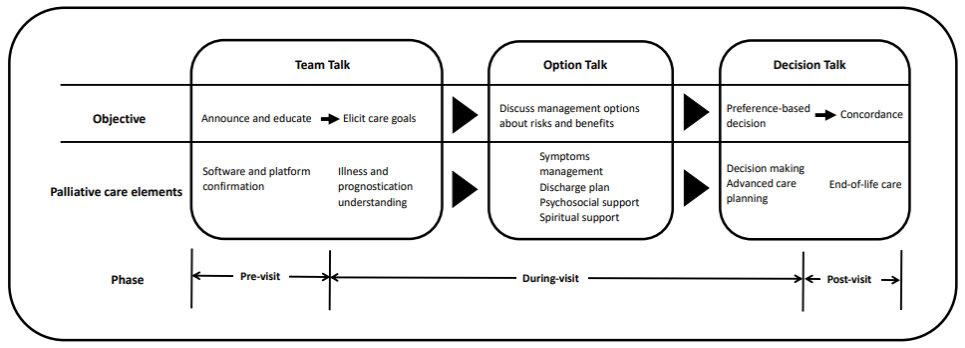
A model for telehealth-based family conferences in palliative care.

A model for telehealth-based family conferences in palliative care was developed by integrating SDM principles like “team talk,” “option talk,” and “decision talk” into the interview structure (Figure 1). The telehealth workflow was implemented into family conferences with previsit, during-visit, and postvisit phases based on the American Medical Association’s Telehealth Implementation Playbook [18]. The availability of telehealth devices and knowledge of the software platform were important requirements for implementation and participation.

The objective of team talk was to assemble the patient, their family, and the multidisciplinary medical team in order to build rapport. In our model, team talk included the previsit phase and the initial part of the during-visit phase. The previsit phase announced the date of the family meeting. Additionally, education on how to use the software and platform on a smartphone was provided to the patient, their family, and the multidisciplinary medical team during this phase. The during-visit phase is marked by the beginning of the telehealth family conference. Moreover, we integrated the palliative care elements of illness and prognostication understanding into the initial stage of this phase. Care goal choices were provided, and the use of the phrase “Shall we describe the options in more detail?” by the physician marked the end of team talk.

Option talk was designed to discuss treatment options, and their associated risks and benefits, with patients and their families. Elements of palliative care such as symptom management and discharge planning were included in option talk. Furthermore, to provide psychosocial and spiritual support, the VALUE approach was emphasized during the reflection and deliberation portion of the telehealth conference. During discussions on the risks and benefits of management options for end-of-life care, such as withdrawal of life-sustaining treatments and discharge planning, possible psychological symptoms in patients like demoralization and distress about dying would induce negative emotions during the conference [19]. The VALUE approach, specifically “value and appreciate what the family member said,” “acknowledge the family member’s emotions,” and “understand who the patient was as a person,” is important in communicating the options.

The aim of decision talk was to reach preference-based decisions on care goals. Advanced care planning during end-of-life care like discharge planning and withdrawal of life-sustaining treatments were the main goals in the model. Using the VALUE approach, we elicited informed preferences through the element “understand the patient as a person” to reach the decision. An important step during this phase is contacting every family member on different devices and interfaces to ensure they are ready to make decisions without network connection barriers. The postvisit phase was also included in the decision talk step, and patient and family experiences toward the conference was evaluated.

### Implementation of the Telehealth Family Conference Model

The telehealth model was implemented in the palliative care unit of the National Taiwan University Hospital, a national cancer treatment center in Taiwan. The hospital set restrictions on family visits and allowed only one caregiver per patient in the inpatient setting during the COVID-19 pandemic. Telehealth-based family conferences were arranged in order to involve additional family members to communicate critical care decisions. The flowchart shown in Figure 1 was implemented after February 2020 in the palliative care unit. Patients admitted to the palliative care ward were eligible to enroll if they were more than 20 years old and required a meeting concerning care goals with the multidisciplinary palliative care team. Families joining the telehealth conference needed to be capable of using a smartphone.

Before the family conference, the date of the meeting was confirmed with the patient and their family. Discussion on which smartphone software or app to use was also carried out prior to the conference. The platform was chosen according to family members’ knowledge and convenience. The interview guide for a video-based family conference was distributed to all medical professionals joining the conference in advance. During the option talk phase, the VALUE approach was integrated into the semistructured meeting. In the decision talk phase, consensus on care goals was achieved, and advanced directives on end-of-life care were documented.

### Data Collection and Analysis

The family conference was arranged when a care goal needed to be communicated to the patient, their family, and the medical teams during the study period. The family conference was summarized and uploaded to the electronic health record system of the hospital. Conversations between the patient, their family, and the multidisciplinary team, which were based on the VALUE approach, were documented. Consensus on care goals was achieved if a decision was made regarding discharge planning, including community-based palliative care or continued hospitalization, and withdrawal of life-sustaining treatments, such as antibiotics or blood transfusion, after the telehealth family conference. Feedback was then evaluated through three questions answered by the patient and their family members together after the conference:

1. Is this your first telehealth family conference?
2. Do you want to use telehealth conferencing again?
3. Do you prefer to talk with your physician face to face?

Family satisfaction toward the meeting was graded on a 5-point scale as follows: 5=very good; 4=good; 3=neutral; 2=bad; and 1=very bad. The analysis was approved by the National Taiwan University Hospital Research Ethics Committee (202004113RINC).

## Results

From February to April 2020 during the COVID-19 pandemic, 14 telehealth family conferences in the palliative care unit were analyzed. The characteristics of the patients are presented in Table 1. The patients’ mean age was 73 (SD 10.1) years, and 6 (43%) patients were female. Among the 14 patients, 12 (86%) were married, and the primary caregiver joining the conference was most frequently a spouse or children (n=10, 71%). The majority of patients (n=13, 93%) were diagnosed with cancer and were terminally ill; 1 (7%) patient was diagnosed with stroke.

Table 1 also shows the number of family members joining the conference using telehealth devices in addition to the one member permitted at bedside. There were 9 (64%) family conferences with more than 2 family members using telehealth software, and 5 (36%) conferences with one family member joining via telehealth.

Two main themes of the family conference include discharge planning and withdrawal of life-sustaining treatments. Only one family meeting to discuss discharge planning did not reach consensus after the conference whereas 8 (89%) did. In terms of conferences on withdrawal of life-sustaining treatment, 100% (n=5) of families reached concordance. In total, consensus was reached in 13 out of 14 conferences (94%).

There were 12 families (90%) using video conferencing for the first time in the health care context, and 10 families (71%) were willing to use video conferencing again for family meetings. However, 7 families (50%) preferred to communicate with medical teams face to face. Level of satisfaction experienced by the families while using video conferencing is demonstrated in Table 2. In total, 5 out of 14 families rated the family meeting as *good* or *very good* (36%), 9 families provided *neutral* feedback (64%), and there was no negative feedback.

**Table 1:** Demographic characteristics of patients (N=14).

Patient		Patients
**Gender, n (%)**		
	Male	8 (57)
	Female	6 (43)
**Age (years), mean (SD)**		73 (10.1)
	Under 40, n (%)	0 (0)
	41-50, n (%)	1 (7)
	51-60, n (%)	1 (7)
	61-70, n (%)	3 (22)
	71-80, n (%)	3 (22)
	Over 80, n (%)	6 (43)
**Marital status, n (%)**		
	Married	12 (86)
	Single	1 (7)
	Separated or divorced	0 (0)
	Widowed	1 (7)
**Education, n (%)**		
	Illiterate	2 (14)
	Elementary school	6 (43)
	Junior high school	3 (21)
	High school	0 (0)
	Bachelor	2 (14)
	Master or PhD	0 (0)
	Unknown	1 (7)
**Primary caregiver, n (%)**		
	Spouse	5 (36)
	Daughter or son	5 (36)
	Sibling	2 (14)
	Other	2 (14)
**Number of family members capable of using telehealth, n (%)**		
	1	5 (36)
	2	4 (29)
	3	2 (14)
	4	2 (14)
	5	1 (7)
**Diagnosis, n (%)**		
	Cancer	13 (93)
	Stroke	1 (7)

**Table 2:** Family attitudes and satisfaction toward telehealth use in palliative care family conferences.

Variable			Participants, n (%)
**Attitudes**			
	**Is this your first time attending a video-based family conference?**		
		Yes	12 (90)
		No	2 (10)
	**Do you want to use video conferencing again?**		
		Yes	10 (70)
		No	4 (30)
	**Do you prefer to talk with your doctor face to face?**		
		Yes	7 (50)
		No	7 (80)
**Family satisfaction**			
	**How do you feel about this telehealth conference compared to face-to-face conferences?**		
		Very good	2 (14)
		Good	3 (22)
		Neutral	9 (64)
		Bad	0 (0)
		Very bad	0 (0)

The 14 family conferences were divided into two groups that were labeled as the neutral group (n=9, 64%) and the satisfied group (rating: *good* and *very good*; n=5, 36%). Categorical variables in Table 3 demonstrate possible factors that relate to respondents’ satisfaction toward telehealth family conferences. A chi-square test did not reveal any statistically significant relationships between the two groups. Further logistic regression analysis also showed no statistically significant associated variables.

**Table 3:** Univariate analysis (χ²) comparing the satisfied group (rating: good and very good) to the neutral group.

Variable		Neutral (n=9), n (%)	Satisfied (n=5), n (%)	χ²	*P* value
**Age**				0.280	.60
	≤65 years	3 (75.0)	1 (25.0)		
	>65 years	6 (60.0)	4 (40.0)		
**Gender**				4.381	.06
	Male	7 (87.5)	1 (12.5)		
	Female	2 (33.3)	4 (66.7)		
**Education**				4.563	.21
	Less than elementary school	6 (75.0)	2 (25.0)		
	High school	2 (66.7)	1 (33.3)		
	Bachelor or higher	0 (0.0)	2 (100.0)		
	Unknown	1 (100.0)	0 (0.0)		
**Marital status**				2.385	.30
	Married	8 (66.7)	4 (33.3)		
	Single	1 (100.0)	0 (0.0)		
	Widowed	0 (0.0)	1 (100.0)		
**Primary caregiver**				4.853	.18
	Spouse	4 (80.0)	1 (20.0)		
	Daughter or son	0 (0.0)	2 (100.0)		
	Sibling	4 (80.0)	1 (20.0)		
	Other	1 (50.0)	1 (50.0)		
**Number of family members using video conferencing**				1.998	.16
	1	2 (40.0)	3 (60.0)		
	>1	7 (77.8)	2 (22.2)		
**Diagnosis**				2.385	.30
	Cancer	8 (66.7)	4 (33.3)		
	Stroke	0 (0.0)	1 (100.0)		

Patients and family members also provided comments about the telehealth model. Some positive comments were as follows:

Telehealth is better than a face-to-face meeting since I don’t need to go to the hospital. I am really anxious about the current COVID-19 threats.

Thank you for your arrangement on this kind of smartphone-enabled communication. I could join the meeting from my office.

There were also some suggestions such as:

My father is a terminal cancer patient! The hospital should relax the infection control measures and let more family members accompany terminal patients in the emotional moments approaching the end of life!

A big screen like that of a computer would be better than a smartphone-enabled model.

Frequent lags, and the network transmission is not smooth! Interruptions occurred several times during physicians’ explanations on the prognosis.

## Discussion

As demonstrated by this study, the telehealth model achieved the aim of enabling more family members to join family conferences under visitor restrictions due to the COVID-19 pandemic. In addition, consensus was achieved on care goals through telehealth communication similar to face-to-face meetings, and high satisfaction toward smartphone-enabled telehealth was seen.

### Increases in Communication Through the Telehealth-Based Family Conference During the COVID-19 Pandemic

Under the COVID-19 pandemic, nearly all hospitals implemented visiting restrictions to reduce personal protective equipment usage due to limited resources and the risk of exposure or nosocomial infection; this included restrictions on visiting hours and the number of visitors [20]. In Taiwan, only one caregiver per patient is permitted to enter the hospital, which increased the difficulty of arranging family conferences. In addition, those who lived abroad were deprived of the opportunity to participate in such events; this also applied to those under home quarantine or home isolation, or following a self-health procedure. Telehealth-based family conferences ameliorated this situation by virtually assembling family members to discuss medical requirements and relieve emotional burdens. With the technology of video software on smartphones, we could now establish clinician-family relationships and facilitate efficient communication with the family even if they are at their workplace or unable to attend in person. Smartphones are so widely used by the general population that it hardly causes any inconvenience to the family and the health care team when used as a venue for conversation. The results of this pilot observational study demonstrated that the number of family members joining the conference increased with the aid of telehealth.

### Integration of SDM in the Telehealth Model to Reach Consensus on End-of-Life Care Goals

Previous studies have shown that family conferences played an indispensable role in facilitating communication with the patient and the family, and further optimized the provision of holistic, goal-concordant care [14,21,22]. However, there has been sparse evidence on the effectiveness of telehealth approaches. In addition, terminally ill patients were often confronted with multiple challenges to conquer and decisions to make, including withdrawal of life-sustaining treatments, prognostication awareness, treatment options, and anticipatory bereavement. Appropriate measurements that comply with SDM can help not only patients but also caregivers in further understanding palliative care, and thus enabling smooth communication with medical professionals. Therefore, our study took advantage of the technology trend and incorporated SDM into the core content of our family conferences, with the aim of helping patients and their families to make optimal decisions for end-of-life management. The results of our study showed that concordance on care goals was high even for difficult decisions like discharge planning and withdrawal of life-sustaining treatments. Thus, this model is feasible for adoption in the palliative care ward during situations like the COVID-19 pandemic, and has the potential to influence regulation on health insurance reimbursement.

### Integration of the VALUE Approach in Palliative Care Family Conferences to Help Achieve Patient and Family Satisfaction With Telehealth

Compared to the traditional face-to-face model, one of the major concerns of telehealth is the physician-patient relationship. There has been some debate that the virtual setting of the venue might make participants miss visual clues, hence, diminishing the quality and goals of the conference, not to mention the lack of adequate physical contact such as a handshake or an assuring patting action as a vital step to establish rapport [23]. Previous studies have revealed that about 7% of emotional communication takes place verbally, while 22% was expressed by tone of voice and 55% by hand gestures, gaze, and eye contact [22]. Therefore, communicating clinical conditions by talking on the phone to the family seems insufficient in building and maintaining rapport with them due to a lack of nonverbal behaviors [24]. There have been studies showing that patients and physicians could bridge the gap in communication and achieve goals effectively through video consultation; however, application to palliative care settings is lacking, where there is much emphasis on face-to-face conversations [25]. Smartphones with relevant apps have played an important role during the COVID-19 pandemic due to social distancing and strict infection control measures [26]. Multidisciplinary medical teams should practice communicating in the telehealth family medicine context using smartphones during the current stringent period. In our model, we implemented the VALUE approach in family meetings, which enabled and encouraged the family to speak more, and made the discussion more rewarding for both sides. Additionally, the family’s emotional cues including (positive) smiles or (negative) frowning were visible on the screen during the meeting. Further studies are needed to explore the influence of these emotional cues on the results of the family conference. The survey on the postvisit phase revealed neutral attitudes or satisfaction toward the current model, and the results indicated that the VALUE approach was suitable for telehealth-based family conferences in palliative care under the current visitor restrictions in hospitals.

### Limitations

Here, we acknowledge a number of limitations pertaining to the study. First, the family conference process was not recorded, and the contents were summarized mainly by medical professionals. Therefore, there may be observational bias in the study. Secondly, the wireless network’s poor performance during some conferences interfered with communication between physicians and family members; this may influence rapport due to waiting times during these disruptions. The restricted availability of the telehealth software also created obstacles to arranging video-based family meetings. Some families preferred to come to the hospital instead since they were reluctant to install the software or learn how to use it. Lastly, the lack of physical contact was another drawback of telehealth meetings, especially in palliative care settings. Among the various goals of arranging family conferences in the model, discerning when and how to facilitate the family in bidding farewell remained the most difficult topic, which required sensitivity not only through dialogue or facial expressions but also through physical gestures (ie, touching).

### Conclusions

During the COVID-19 pandemic, health care professionals must adhere to the restrictions implemented for transmission prevention. A telehealth model for family conferences in palliative care with SDM and VALUE integration demonstrated high satisfaction in family members and was effective in reaching consensus about care decisions. The model may be applied to other countries to promote quality in end-of-life care in the era of COVID-19.

## Abbreviations

ICU: intensive care unit
SDM: shared decision making
VALUE: value, acknowledge, listen, understand, and elicit
